# Reply to: Letter to the editor of Heliyon re design and optimization of a high efficiency CdTe–FeSi2 based double-junction two-terminal tandem solar cell. Heliyon. 10 (2024) e27994

**DOI:** 10.1016/j.heliyon.2024.e29388

**Published:** 2024-04-16

**Authors:** Jaker Hossain

**Affiliations:** Department of Electrical and Electronic Engineering, University of Rajshahi, Rajshahi, 6205, Bangladesh

Dear Editor,

We are grateful for Dr. Alexander P. Kirk's interest and thorough analysis on our article. Every comment has been addressed here and they have proven to be incredibly helpful in shaping our understanding.

In our work, we have utilized a range of mathematical and physical principles to craft and enhance the efficiency of CdTe–FeSi_2_ based double-junction two-terminal tandem solar cell. We have employed Poisson's equations to model the electric potential within the solar cell, crucial for comprehending the electron and hole movement driven by electric fields. Our selection of CdTe and FeSi_2_ as materials for the top and bottom cells, respectively, stems from meticulous bandgap engineering, aiming to absorb a broad spectrum of solar radiation effectively. Optical filtering has been implemented to ensure appropriate absorption of each part of the solar spectrum by the respective cell layers. Through mathematical optimization, we have fine-tuned various parameters such as layer thickness and doping levels to maximize solar cell efficiency. Additionally, we have conducted an in-depth analysis of defects across different layers, recognizing their significant influence on solar cell performance. Spectral response analysis has likely been carried out to assess the cell's responsiveness to various solar spectrum wavelengths, offering valuable insights for further enhancements. This overview encapsulates the key mechanisms integral to our work.

Dr. Kirk asserted that the simulated values surpass the theoretical limits of power conversion efficiency, open circuit voltage, and fill factor for a 1.5 eV/0.87 eV 2-junction solar cell as dictated by detailed balance principles. According to detailed balance analysis, the maximum theoretical upper efficiency limit for an ideal 2T dual-junction (2J) solar cell was estimated to be between 42 % and 46 % [[1], [2], [3], [4]]. Dr. Kirk's analysis of the results reveals three distinct cases for the CdTe/FeSi_2_ double-junction solar cell. In Case 1, the CdTe sub-cell exhibits a photo-current density (J_SC_) of 25.743 mA/cm^2^, a photo-generated open circuit voltage (V_OC_) of 1.2289 V and the FeSi_2_ sub-cell demonstrates a J_SC_ of 26.343 mA/cm^2^, a V_OC_ of 0.6277 V. The total fill factor (FF) of 88.02 %, resulting in an efficiency (η) of 42.1 %. Similarly, in Case 2, J_SC_ for both sub-cells remains consistent, resulting in an efficiency of 41.6 %. Case 3 demonstrates slightly lower performance metrics compared to Cases 1 and 2, with a J_SC_ of 25.340 mA/cm^2^ and an efficiency of 40.4 %. On the contrary, we have achieved V_OC_ of 1.181 V and J_SC_ of 25.13 mA/cm^2^ for the top cell, and V_OC_ of 0.726 V and J_SC_ of 25.13 mA/cm^2^ for the bottom cell, with an FF of 89 % and efficiency of 43.9 %. Clearly, the cause behind the deviant on the result is due to the performance variation of bottom cell, as the performance of top cell is quite similar in both cases. The primary cause of these discrepancies lies in the selection of different mechanisms; while we utilized filtered spectrum, by using [5].

(1) $${S\left(\lambda \right)=S}_{0}\;\exp\;\left(\sum_{i}^{n}-\left(a\left(\lambda \right)\times d_{{mat}_{i}}\right)\right)$$

where the incoming AM 1.5 G is mentioned as S_0_(λ) and modified spectrum as S(λ), respectively.

Dr. Kirk implemented a tunnel junction between the top and bottom cell. The ideal bandgap pairing and the maximum achievable efficiency as documented in existing literature exhibit slight variations, influenced by the specific model and assumptions applied during calculations [6]. When utilizing a filtered spectrum for the bottom cell, optimization to absorb a specific solar spectrum range may enhance efficiency. However, the inclusion of a tunnel junction connecting the top and bottom cells has potential implications for efficiency. Tunnel junctions, serving as conductive and optically transparent semiconductor layers to join different materials, have a crucial impact on tandem cell efficiency. It is imperative that the tunnel junction remains transparent to allow light transmission to the bottom cell; excessive absorption by the tunnel junction could diminish the amount of light reaching the bottom cell, thus affecting its efficiency [7]. In our design case, we assumed an ideal tunnel junction.

Furthermore, Dr. Kirk has noted that we have indicated our expectation for our CdTe/FeS_2_ solar cell to achieve a fill factor of 89.88 %. This projection implies a substantial enhancement in fill factor; however, no discussion has been provided on how such a considerable improvement has been realized in an actual device. The FF is related with V_OC_ by following equation [8],

$$\text{FF}=\frac{v_{oc-\ln\left(v_{oc}-0.72\right)}}{v_{oc}}$$

According to this equation, if we put V_OC_ = 1.92, we get FF equals to 90 % which is very close to our FF = 89.8 %.

Dr. Kirk has also pointed out that, besides, we have not quantified the realistic reduction in current density resulting from top grid electrode coverage, imperfections in the anti-reflection coating, or parasitic absorption in the tunnel junction. While optimizing the spectrum filtering may indirectly affect overall absorption characteristics and mitigate some parasitic absorption, it does not specifically target or quantify the absorption occurring within the tunnel junction itself.

It has been observed that when a thickness of 550 nm was fixed for the CdTe top sub-cell, the short circuit current density J_SC_ in the FeSi_2_ bottom sub-cell has remained constant even as its thickness has been varied from 100 nm to 700 nm. This phenomenon may be due to the absorption of incoming photons deep within the active layer, diverting them from the depletion zone. Consequently, the newly formed electron-hole pair is unable to enter the space charge area during its lifetime, resulting in bulk recombination of the carrier, as previously indicated [9].

Additionally, according to Dr. Kirk, We have claimed that large acceptor doping concentration values of 10^18^ cm^−3^ in the CdTe absorber and 10^21^ cm^−3^ in the FeSi_2_ absorber yield the highest power conversion efficiency. However, it is acknowledged that such high doping concentrations are known to adversely affect minority carrier lifetime and, consequently, device efficiency. We have optimized the doping concentration of the top layer to 10^16^ cm^−3^ and for the bottom layer to 10^18^ cm^−3^. Interestingly, with higher doping in the top cell, a slight decrease in efficiency is observed, while for the bottom cell, a slight enhancement is noticed. This observation suggests that higher doping may lead to a semiconductor exhibiting metallic characteristics.

In conclusion, our CdTe/FeSi_2_ double-junction solar cell demonstrates performance within theoretical limits, albeit with variations across different cases. However, discrepancies with Dr. Kirk's findings arise due to the use of different mechanisms.

## CRediT authorship contribution statement

**Jaker Hossain:** Writing – review & editing, Writing – original draft, Sheikh Noman Shiddique, Writing – original draft, Ahnaf Tahmid Abir, Writing – original draft, Mehedi Hasan Tonmoy, Writing – original draft.

## Declaration of competing interest

The authors declare that they have no known competing financial interests or personal relationships that could have appeared to influence the work reported in this paper.
